# Supplementary material for: N1-methyl-pseudouridine is incorporated with higher fidelity than pseudouridine in synthetic RNAs
Source: Sci Rep. 2022 Jul 29;12:13017. doi: 10.1038/s41598-022-17249-1 (PMC9335462; doi:10.1038/s41598-022-17249-1)
Supplement: Supplementary file 1 — Supplementary Information. [file 41598_2022_17249_MOESM1_ESM.pdf]

N^1-methyl-pseudouridine is incorporated with higher fidelity than pseudouridine in synthetic RNAs.

Tien-Hao Chen, Vladimir Potapov, Nan Dai, Jennifer L. Ong, Bijoyita Roy*

RNA and Genome Editing, New England Biolabs Inc, Beverly, MA

*Corresponding author:

Bijoyita Roy

Email: broy@neb.com

Supplementary Information

Supplementary Figures

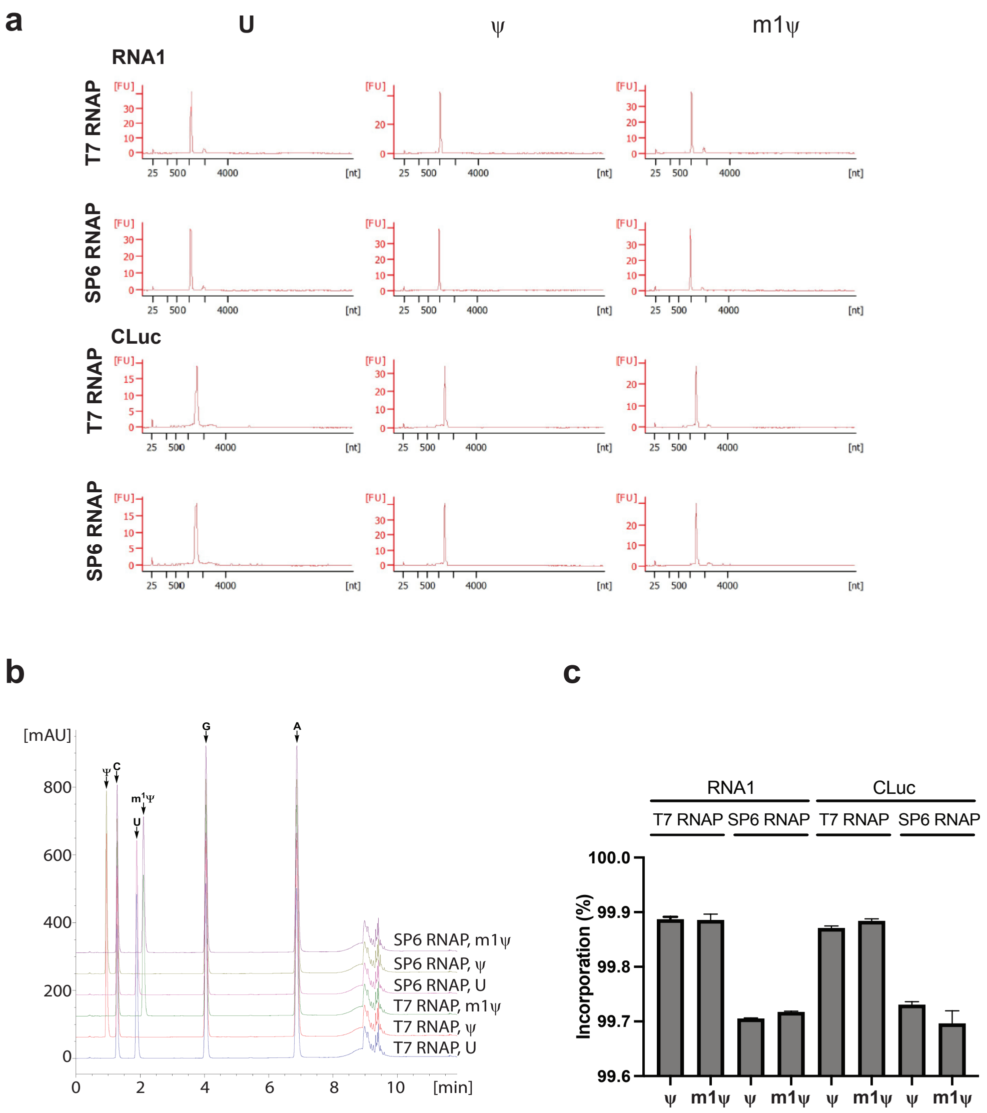

Supplemental Figure 1. **T7 and SP6 RNA polymerases incorporate U, ψ, and m1ψ into full-length RNAs. a.** Representative bioanalyzer traces of RNA1 and CLuc transcribed with uridine analogs showing synthesis of the full-length RNA and the integrity of the RNA. **b.** Representative UHPLC traces for RNA1. The purified RNA digested with a nucleoside digestion mix was analyzed. **c.** Incorporation of uridine analogs in RNA1 and CLuc assessed using UHPLC/MS. All the ψ- and m1ψ-modified transcripts have higher than 99.6% incorporation of modified uridine. Data reported are an average of three independent experiments.

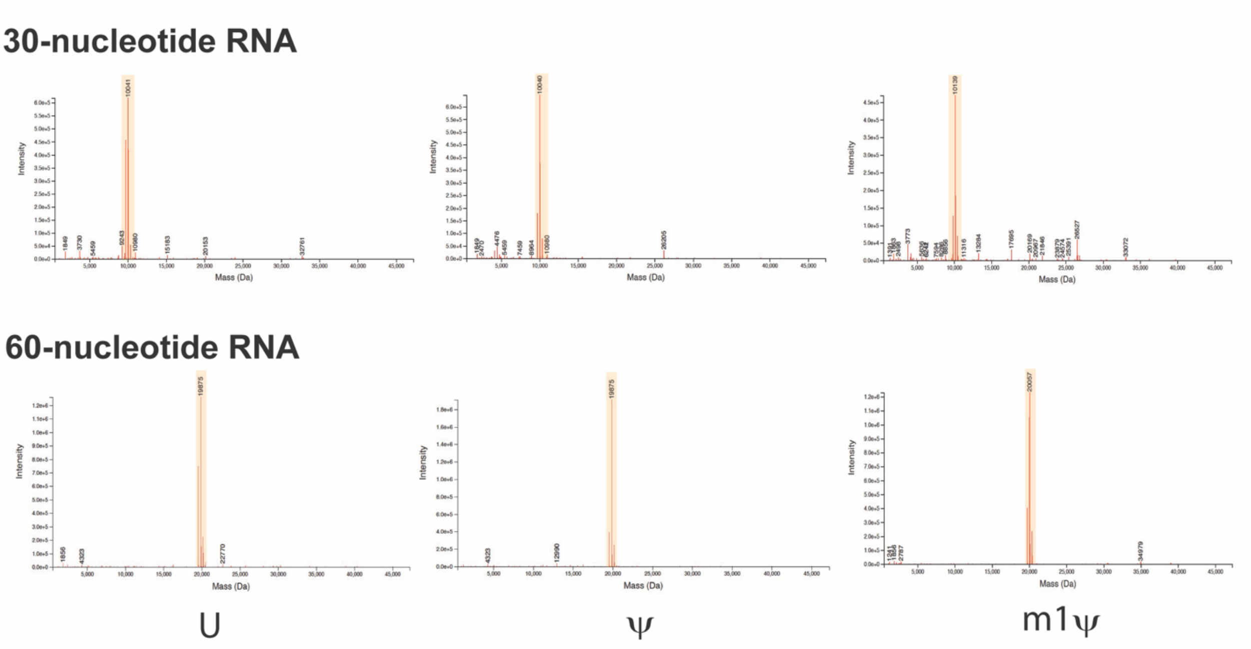

Supplemental Figure 2. T7 RNA polymerase synthesizes run-off products and run-off products with non-templated additions in presence of U, ψ, and m1ψ. Short 30-nucleotide (30-nt) RNA and 60-nucleotide (60-nt) RNA were transcribed *in vitro* and assessed using intact mass spectrometry. From the top four abundant masses, the RNAs that were either transcribed as expected or with one or two non-templated nucleotide additions are highlighted in the orange box as run-off transcripts. Data was analyzed using the ProMass deconvolution software from Novatia, LLC.

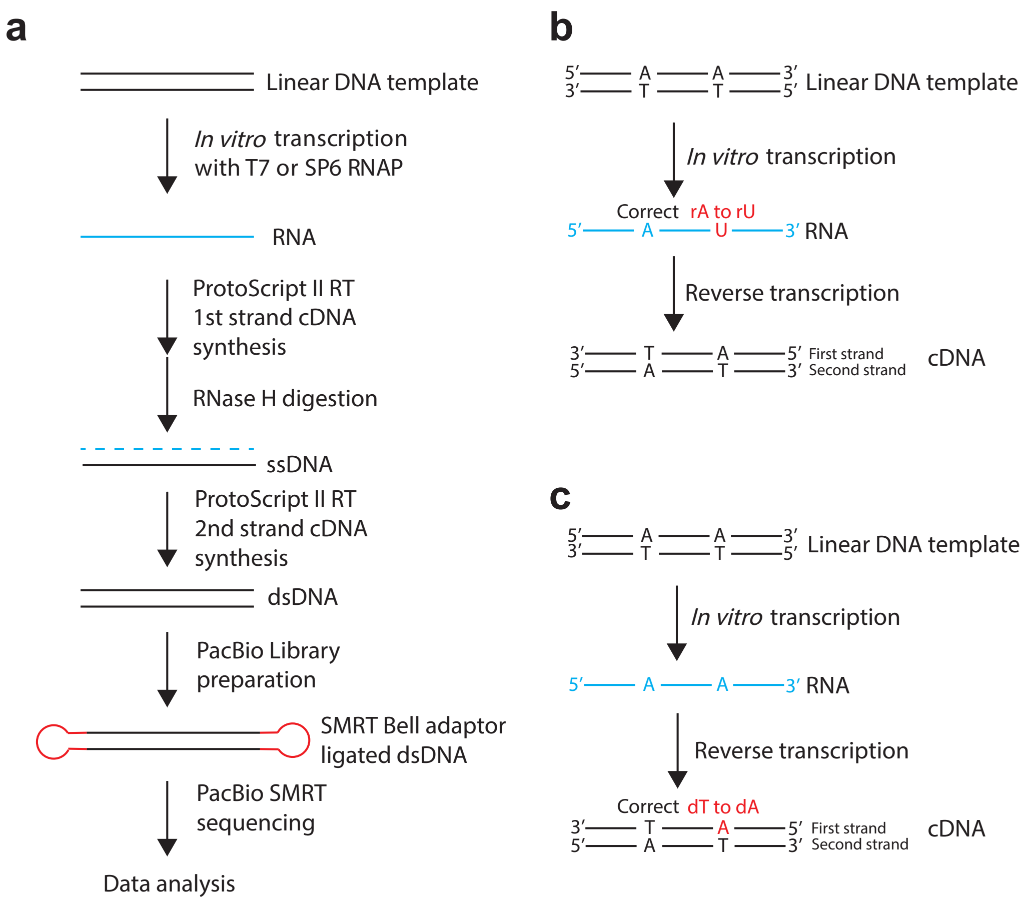

Supplemental Figure 3. Schematic illustration of workflow to measure combined fidelity of RNA polymerases and ProtoScript II reverse transcriptase. **a.** Linear dsDNA served as a template to synthesize RNA with unmodified and modified uridine by T7 or SP6 RNA polymerase. The transcript was reverse transcribed into first strand and second strand cDNA by ProtoScript II reverse transcriptase. The dsDNA was ligated with SMRTbell adaptor and subjected for sequencing on the PacBio system. Substitution errors observed (for example an rA→rU/dT→dA) can occur either during *in vitro* transcription or reverse transcription. **b.** During *in vitro* transcription, RNA polymerase might mis-incorporate an rU (red) in place of rA. The correct incorporation of rA (red) and the resulting cDNA were shown. **c.** During the first strand cDNA synthesis, reverse transcriptase might mis-incorporate a dT (red) to the position of dA. The correct incorporation of dA and the resulting cDNA were shown.

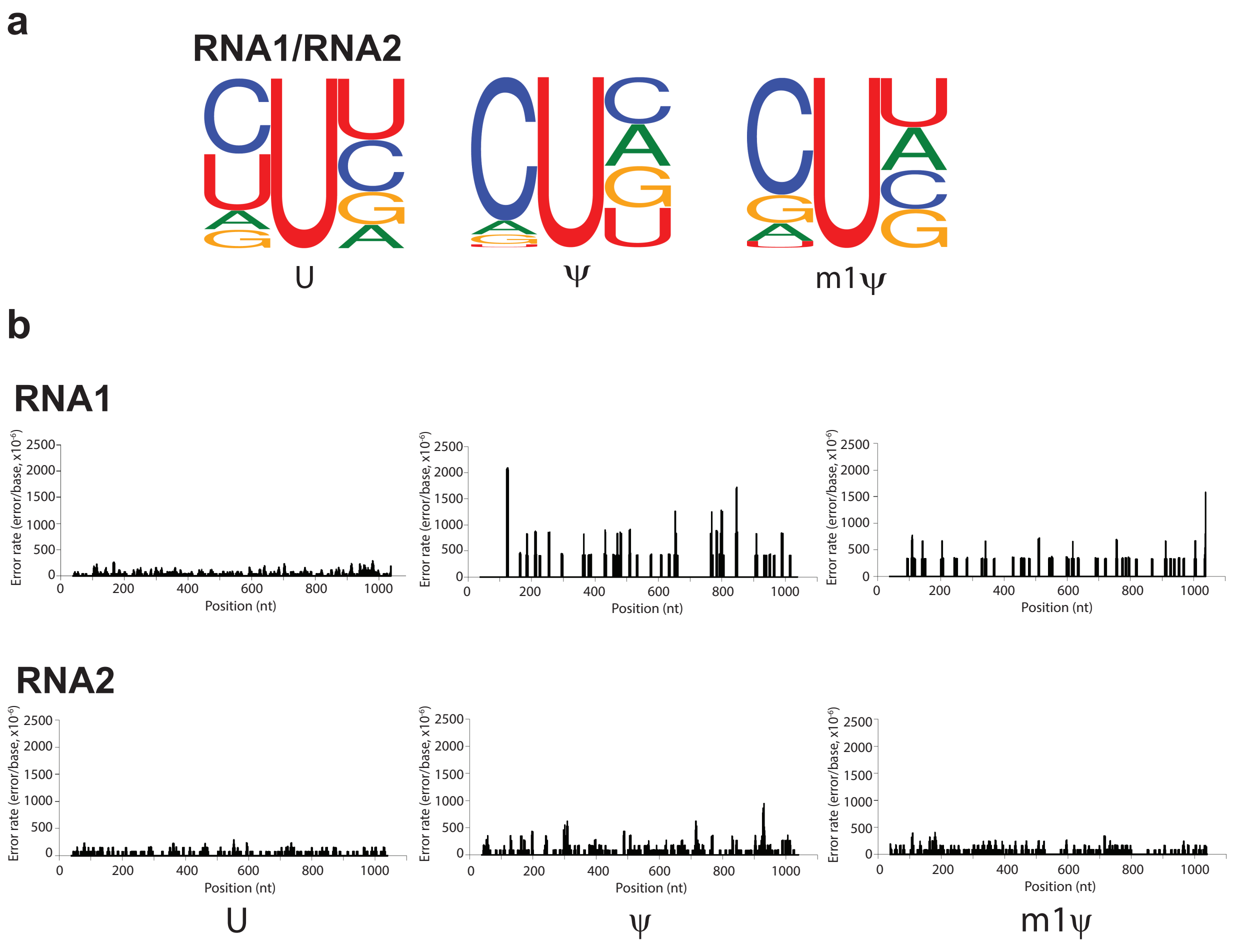

Supplemental Figure 4. rA→rU/dT→dA substitution errors observed in ψ–containing RNAs have sequence context preference and occur throughout the transcript body when reactions are performed with T7 RNA polymerase. The data was generated from two artificial RNA sequences (RNA1 and RNA2 with sequence permutation to include every four-base combination). **a.** Logo of sequence surrounding the rA→rU/dT→dA substitution sites. Seven nucleotides upstream and downstream of the substitution site were examined and for simplicity the immediate sequence upstream and downstream of the substitution site is represented. Sequence logos were built using WebLogo software[1] and the numbers of sites plotted are summarized in Supplementary Table 15. **b.** Substitution error distribution along the length of the transcript.

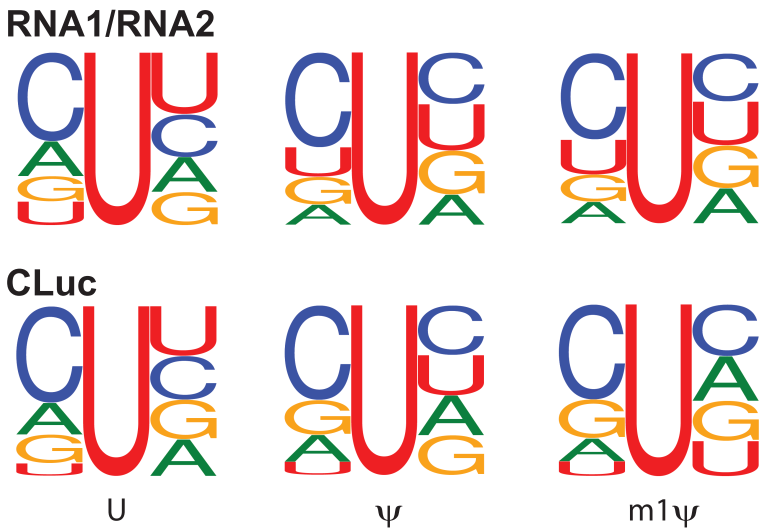

Supplemental Figure 5. rA→rU/dT→dA substitution errors observed in RNAs synthesized with SP6 RNA polymerase do not have sequence context preference. The sequence surrounding the rA→rU/dT→dA substitution sites were used to generate the sequence logo. The data was generated from two artificial RNA sequences (RNA1 and RNA2 with sequence permutation to include every four-base combination) and CLuc mRNA. Seven nucleotides upstream and downstream of the substitution site were examined and for simplicity the immediate sequence upstream and downstream of the substitution site is represented. Sequence logos were built using WebLogo software[1] and the numbers of sites plotted are summarized in Supplementary Table 15.

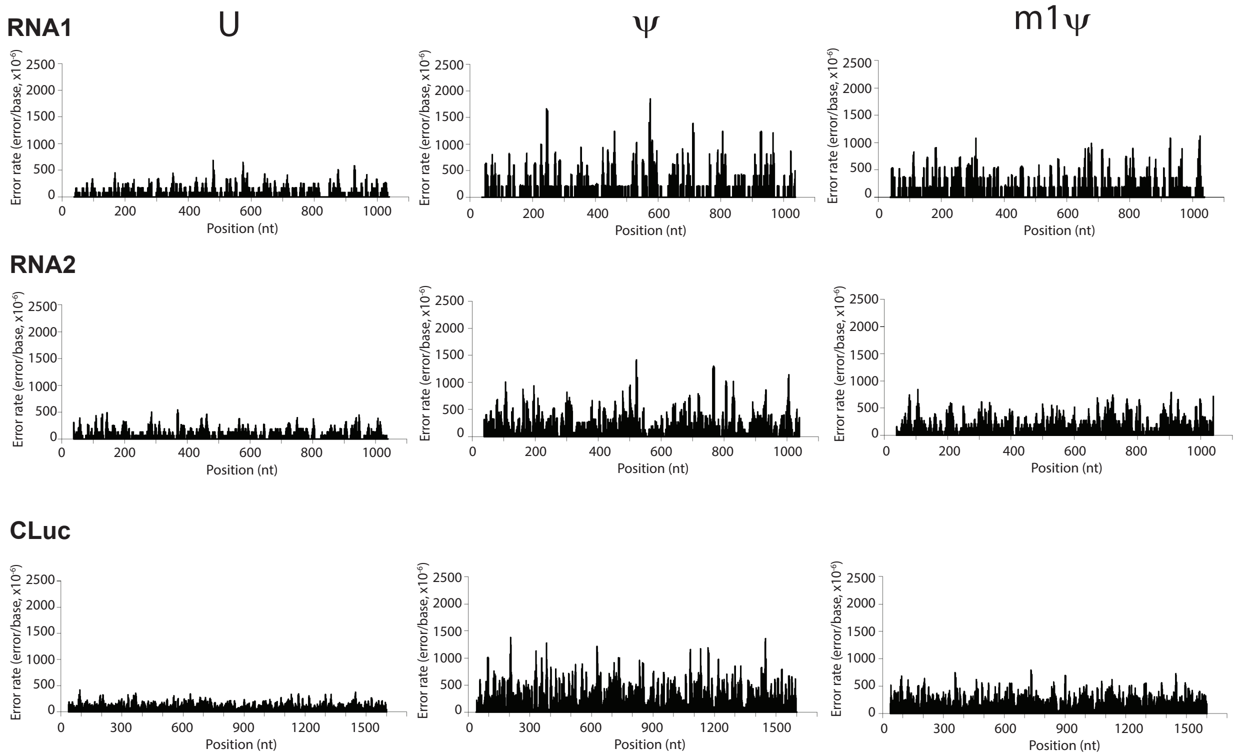

Supplemental Figure 6. rA→rU/dT→dA substitution errors are observed along the length of the RNA with no preference for a specific position in the transcript when reactions are performed with SP6 RNA polymerase. Substitution errors in three RNAs were assessed: artificial RNA1 and RNA2 with sequence permutation to include every 4-base combination, and a functional CLuc mRNA.

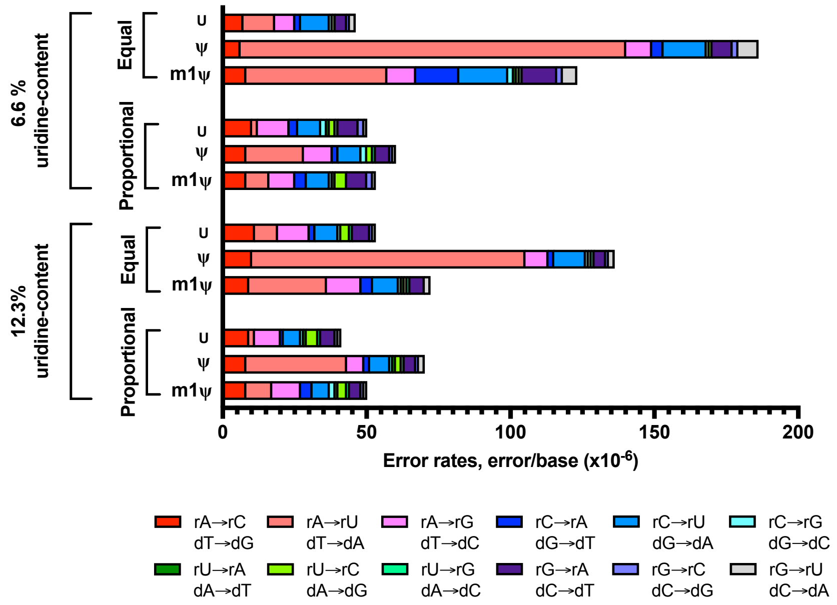

Supplemental Figure 7. *In vitro* transcription error profile of T7 RNA polymerase can be modulated by altering ribonucleotide composition in the reaction. Uridine-depleted artificial RNA sequences (with 6.6% or 12.3% uridine content) were transcribed with T7 RNA polymerase and reactions were performed with either equal molar rNTPs or rNTPs proportional to the template sequence. Base substitution error profile observed for unmodified and modified RNA sequences from reactions under the two different rNTP reaction conditions are represented. Colors indicate specific substitution errors observed.

Supplementary Tables

Supplementary Table 1. Comparison of combined error rates for unmodified RNA1/RNA2 synthesized with T7 RNA polymerase measured using either RSII or Sequel platform.

Platform	Total error rate ($\times 10^{-6}$, errors/base)	Substitution (%)	Deletion (%)	Insertion (%)	Total sequenced bases	Reference
RSII	56 ± 8	71	19	10	30,868,961	Potapov *et al.*[2]
RSII	41 ± 4	80	11	9	12,024,831	This study
Sequel	64 ± 4	73	12	15	7,820,043	This study

Supplementary Table 2. Substitution error rates of first strand cDNA observed in RNA1/RNA2 (combined; n=1 for RNA1 and RNA2), CLuc (n=2) and BNT162b2 (n=2) mRNAs synthesized with T7 RNA polymerase. The relative fold change was calculated for each substitution as (M—U) / U, where M is the substitution rate on modified RNA and U is the substitution rate on unmodified RNA. N/A, not applicable, as the error rates were rounded to zero in unmodified RNA.

**RNA**	**Uridine analog**	**Substitution error rate ($\times 10^{-6}$, errors/base)**											
		**rA→rC dT→dG**	**rA→rU dT→dA**	**rA→rG dT→dC**	**rC→rA dG→dT**	**rC→rU dG→dA**	**rC→rG dG→dC**	**rU→rA dA→dT**	**rU→rC dA→dG**	**rU→rG dA→dC**	**rG→rA dC→dT**	**rG→rC dC→dG**	**rG→rU dC→dA**
RNA1/ RNA2	U	4	5	7	2	7	1	1	8	2	8	1	2
	ψ	4	58	4	8	7	0	3	4	1	5	0	4
	m1ψ	8	15	10	5	7	0	2	7	1	9	1	1
	**Relative fold change (ψ to U)**	**0**	**11**	**0**	**3**	**0**	**-1**	**2**	**-1**	**-1**	**0**	**-1**	
	**Relative fold change (m1ψ to U)**	**1**	**2**	**0**	**2**	**0**	**-1**	**1**	**0**	**-1**	**0**	**0**	**-1**
CLuc mRNA	U	11	10	11	1	8	0	1	7	1	2	1	1
	ψ	7	98	9	2	7	1	1	4	1	3	1	4
	m1ψ	8	23	14	6	9	0	4	4	2	3	0	0
	**Relative fold change (ψ to U)**	**0**	**9**	**0**	**1**	**0**	**N/A**	**0**	**0**	**0**	**1**	**0**	**3**
	**Relative fold change (m1ψ to U)**	**0**	**1**	**0**	**5**	**0**	**N/A**	**3**	**0**	**1**	**1**	**-1**	**-1**
BNT162b2	U	4	6	6	1	11	0	1	5	1	5	1	1
	ψ	3	79	7	1	14	1	0	3	1	6	2	3
	m1ψ	7	22	5	3	9	1	2	5	1	5	1	1
	**Relative fold change (ψ to U)**	**0**	**12**	**0**	**0**	**0**	**N/A**	**-1**	**0**	**0**	**0**	**1**	**2**
	**Relative fold change (m1ψ to U)**	**1**	**3**	**0**	**2**	**0**	**N/A**	**1**	**0**	**0**	**0**	**0**	**0**

Supplementary Table 3. Total and specific error rates of first strand cDNA observed in unmodified RNA1/RNA2 (combined) synthesized with T3, T7 or SP6 RNA polymerases. The mean and standard deviation were calculated from two independent measurements including one for RNA1 and one for RNA2.

RNA polymerase	Total error rate ($\times 10^{-6}$, errors/base)	Substitution (%)	Deletion (%)	Insertion (%)	Total sequenced bases
T3	68 ± 10	71	9	20	10,981,293
T7	64 ± 4	73	12	15	7,820,043
SP6	140 ± 3	84	6	10	5,463,829

Supplementary Table 4. Total and specific error rates of first strand cDNA in RNA sequences synthesized with SP6 RNA polymerase. Total error rates observed in three different RNA sequences (RNA1, RNA2, and CLuc mRNA) were pooled. The mean and standard deviation were calculated from four independent measurements (n=1 for RNA1, n=1 for RNA2, n=2 for CLuc mRNA).

**Base**	**Total error rate** (×10^{-6}, **errors/base)**	**Substitution (%)**	**Deletion (%)**	**Insertion (%)**	**Total sequenced bases**
U	133 ± 5	89	7	4	38,647,641
ψ	325 ± 20	94	3	3	11,052,338
m1ψ	253 ± 2	93	5	2	38,709,112

Supplementary Table 5. Substitution error rates of first strand cDNA observed in RNA1/RNA2 (combined; n=1 for RNA1 and RNA2) and CLuc mRNA (n=2) synthesized with SP6 RNA polymerase. The relative fold change was calculated for each substitution as (M—U) / U, where M is the substitution rate on modified RNA and U is the substitution rate on unmodified RNA.

**RNA**	**Uridine analog**	**Substitution error rate (×10^{-6}, errors/base)**											
		**rA→rC dT→dG**	**rA→rU dT→dA**	**rA→rG dT→dC**	**rC→rA dG→dT**	**rC→rU dG→dA**	**rC→rG dG→dC**	**rU→rA dA→dT**	**rU→rC dA→dG**	**rU→rG dA→dC**	**rG→rA dC→dT**	**rG→rC dC→dG**	**rG→rU dC→dA**
RNA1/ RNA2	U	14	20	24	3	15	1	2	13	1	16	2	6
	ψ	18	163	26	2	25	1	4	11	2	13	1	10
	m1ψ	28	69	47	6	17	3	5	18	3	20	1	6
	**Relative fold change (ψ to U)**	**0**	**7**	**0**	**0**	**1**	**0**	**1**	**0**	**1**	**0**	**-1**	**1**
	**Relative fold change (m1ψ to U)**	**1**	**2**	**1**	**1**	**0**	**2**	**2**	**0**	**2**	**0**	**-1**	**0**
CLuc	U	23	19	32	1	11	1	2	15	2	6	1	5
	ψ	28	189	37	2	26	1	3	17	1	9	1	7
	m1ψ	31	84	54	2	16	3	4	21	3	12	1	5
	**Relative fold change (ψ to U)**	**0**	**9**	**0**	**1**	**1**	**0**	**1**	**0**	**-1**	**1**	**0**	**0**
	**Relative fold change (m1ψ to U)**	**0**	**3**	**1**	**1**	**0**	**2**	**1**	**0**	**1**	**1**	**0**	**0**

Supplementary Table 6. Total and specific error rates of first strand cDNA observed in RNA sequences synthesized with T7 RNA polymerase under varying rNTP concentrations. Total error rates observed in two different RNA sequences (RNA1 and RNA2) were pooled. The mean and standard deviation were calculated from two independent measurements (n=1 for RNA1 and RNA2).

**rNTP (nM)**	**Base**	**Total error rate (×10^{-6}, errors/base)**	**Substitution (%)**	**Deletion (%)**	**Insertion (%)**	**Total sequenced bases**
10	U	66 ± 9	78	10	12	9,406,619
	ψ	160 ± 1	93	3	5	14,307,731
	m1ψ	120 ± 10	89	6	5	9,202,572
20	U	66 ± 2	77	10	13	16,735,875
	ψ	190 ± 14	93	3	4	13,483,086
	m1ψ	130 ± 4	91	4	5	9,983,664
40	U	64 ± 4	73	12	15	7,820,043
	ψ	110 ± 20	91	4	5	2,764,788
	m1ψ	80 ± 3	84	9	7	3,097,951

Supplementary Table 7. Total and specific error rates of first strand cDNA in RNA sequences synthesized with T7 RNA polymerase under equal or proportional rNTP condition. Total error rates observed in four different uridine-depleted RNA sequences (RNA with 5.5%, 6.6%, and 12.3% uridine content, and uridine-depleted CLuc mRNA) were pooled. The mean and standard deviation were calculated from eight independent measurements (n=2 for each sequence).

**rNTP**	**Base**	**Total error rate** ($\times 10^{-6}$, **errors/base)**	**Substitution (%)**	**Deletion (%)**	**Insertion (%)**	**Total sequenced bases**
Equal	U	60 ± 11	79	15	6	50,069,987
Equal	ψ	190 ± 37	94	4	2	68,391,278
Equal	m1ψ	99 ± 30	89	8	4	78,904,619
Proportional	U	56 ± 21	73	22	6	65,366,664
Proportional	ψ	84 ± 9	87	8	5	71,885,977
Proportional	m1ψ	63 ± 5	83	9	7	70,807,134

Supplementary Table 8. Representative yield from *in vitro* transcription reactions performed with T7 RNA polymerase with either equal molar rNTPs or rNTPs proportional to the template sequence (RNA sequence with 5.5% uridine content).

rNTP	Base	Yield (μg)
Equal	U	80
Equal	ψ	68
Equal	m1ψ	50
Proportional	U	44
Proportional	ψ	70
Proportional	m1ψ	64

Supplementary Table 9. Substitution error rates of first strand cDNA observed in three uridine-depleted RNA sequences (with either 5.5%, 6.6%, or 12.3% uridine content) synthesized with T7 RNA polymerase. Two independent experiments were performed for each RNA sequence. The relative fold change was calculated for each substitution as (M—U) / U, where M is the substitution rate on modified RNA and U is the substitution rate on unmodified RNA. E, Equal molar rNTPs; P, proportional molar ratio of rNTPs to the template sequence.

Template Uridine content	rNTP	Uridine analog	Substitution error rate (×10^{-6}, errors/base)											
			rA→rC dT→dG	rA→rU dT→dA	rA→rG dT→dC	rC→rA dG→dT	rC→rU dG→dA	rC→rG dG→dC	rU→rA dA→dT	rU→rC dA→dG	rU→rG dA→dC	rG→rA dC→dT	rG→rC dC→dG	rG→rU dC→dA
**5.5%**	**E**	U	7	9	10	2	10	1	0	1	0	4	1	1
		ψ	8	131	9	2	13	0	0	1	0	4	1	5
		m1ψ	9	38	9	3	9	1	0	0	0	4	1	2
		**Relative fold change (ψ to U)**	**0**	**14**	**0**	**0**	**0**	**-1**	**N/A**	**0**	**N/A**	**0**	**0**	**4**
		**Relative fold change (m1ψ to U)**	**0**	**3**	**0**	**1**	**0**	**0**	**N/A**	**-1**	**N/A**	**0**	**0**	**1**
	**P**	U	11	2	8	1	7	0	1	3	0	5	1	0
		ψ	9	18	9	1	8	1	1	3	0	4	1	1
		m1ψ	10	5	11	3	7	1	1	3	1	4	1	0
		**Relative fold change (ψ to U)**	**0**	**8**	**0**	**0**	**0**	**N/A**	**0**	**0**	**N/A**	**0**	**0**	**N/A**
		**Relative fold change (m1ψ to U)**	**0**	**2**	**0**	**2**	**0**	**N/A**	**0**	**0**	**N/A**	**0**	**0**	**N/A**
**6.6%**	**E**	U	7	11	7	2	10	1	0	1	0	4	1	2
		ψ	6	134	9	4	15	1	0	1	0	7	2	7
		m1ψ	8	49	10	15	17	2	1	1	1	12	2	5
		**Relative fold change (ψ to U)**	**0**	**11**	**0**	**1**	**1**	**0**	**N/A**	**0**	**N/A**	**1**	**1**	**3**
		**Relative fold change (m1ψ to U)**	**0**	**3**	**0**	**7**	**1**	**1**	**N/A**	**0**	**N/A**	**2**	**1**	**2**

	**P**	U	10	2	11	3	8	2	1	2	1	7	2	1
		ψ	8	20	10	2	8	2	0	2	1	5	1	1
		m1ψ	8	8	9	4	8	1	1	4	0	7	2	1
		**Relative fold change (ψ to U)**	**0**	**9**	**0**	**0**	**0**	**0**	**-1**	**0**	**0**	**0**	**-1**	**0**
		**Relative fold change (m1ψ to U)**	**0**	**3**	**0**	**0**	**0**	**-1**	**0**	**1**	**-1**	**0**	**0**	**0**
**12.3%**	**E**	U	11	8	11	2	8	1	0	3	1	6	1	1
		ψ	10	95	8	2	11	1	0	1	1	4	1	2
		m1ψ	9	27	12	4	9	1	1	1	1	5	0	2
		**Relative fold change (ψ to U)**	**0**	**11**	**0**	**0**	**0**	**0**	**N/A**	**-1**	**0**	**0**	**0**	**1**
		**Relative fold change (m1ψ to U)**	**0**	**2**	**0**	**1**	**0**	**0**	**N/A**	**-1**	**0**	**0**	**-1**	**1**
	**P**	U	9	2	9	1	6	1	1	4	1	5	1	1
		ψ	8	35	6	2	7	1	1	2	1	4	1	2
		m1ψ	8	9	10	4	6	2	1	3	1	4	1	1
		**Relative fold change (ψ to U)**	**0**	**17**	**0**	**1**	**0**	**0**	**0**	**-1**	**0**	**0**	**0**	**1**
		**Relative fold change (m1ψ to U)**	**0**	**4**	**0**	**3**	**0**	**1**	**0**	**0**	**0**	**0**	**0**	**0**

Supplementary Table 10. Substitution error rates of first strand cDNA observed in uridine-depleted CLuc mRNA (n=2) synthesized with T7 RNA polymerase. The relative fold change was calculated for each substitution as (M—U) / U, where M is the substitution rate on modified RNA and U is the substitution rate on unmodified RNA. N/A, not applicable, as the error rates were rounded to zero in unmodified RNA.

rNTP	Uridine analog	Substitution error rate ($\times10^{-6}$, errors/base)											
		**rA→rC dT→dG**	**rA→rU dT→dA**	**rA→rG dT→dC**	**rC→rA dG→dT**	**rC→rU dG→dA**	**rC→rG dG→dC**	**rU→rA dA→dT**	**rU→rC dA→dG**	**rU→rG dA→dC**	**rG→rA dC→dT**	**rG→rC dC→dG**	**rG→rU dC→dA**
Equal	U	7	8	6	1	9	0	0	3	0	5	1	3
	ψ	7	133	6	1	15	0	1	2	1	4	1	12
	m1ψ	7	33	6	4	8	0	1	2	0	8	1	3
	**Relative fold change (ψ to U)**	**0**	**16**	**0**	**0**	**1**	**N/A**	**N/A**	**0**	**N/A**	**0**	**0**	**3**
	**Relative fold change (m1ψ to U)**	**0**	**3**	**0**	**3**	**0**	**N/A**	**N/A**	**0**	**N/A**	**1**	**0**	**0**
Proportional	U	10	4	6	1	6	0	1	4	0	4	1	1
	ψ	9	47	6	1	8	0	2	6	1	4	1	3
	m1ψ	11	12	7	2	6	0	3	8	1	6	1	1
	**Relative fold change (ψ to U)**	**0**	**11**	**0**	**0**	**0**	**N/A**	**1**	**1**	**N/A**	**0**	**0**	**2**
	**Relative fold change (m1ψ to U)**	**0**	**2**	**0**	**1**	**0**	**N/A**	**2**	**1**	**N/A**	**1**	**0**	**0**

Supplementary Table 11. Total and specific error rates of first strand cDNA observed in uridine-depleted RNA sequence (5.5% uridine content) synthesized with SP6 RNA polymerase.

rNTP	Base	Total error rate ($\times 10^{-6}$, errors/base)	Substitution (%)	Deletion (%)	Insertion (%)	Total sequenced bases
Equal	U	281 ± 86	92	4	4	10,587,337
Equal	ψ	597 ± 210	96	2	2	17,735,541
Equal	m1ψ	407 ± 95	94	3	3	7,454,925
Proportional	U	131 ± 1	87	9	5	12,731,671
Proportional	ψ	172 ± 1	91	5	4	26,186,535
Proportional	m1ψ	154 ± 2	88	6	6	7,985,264

Supplementary Table 12. Substitution error rates of first strand cDNA observed in uridine-depleted RNA with 5.5% uridine content synthesized by SP6 RNA polymerase. The fold change between modified and unmodified RNA is highlighted in red. The relative fold change was calculated for each substitution as (M—U) / U, where M is the substitution rate on modified RNA and U is the substitution rate on unmodified RNA. N/A, not applicable, as the error rates were rounded to zero in unmodified RNA.

**rNTP**	**Uridine analog**	**Substitution error rate ($\times 10^{-6}$, errors/base)**											
		**rA→rC dT→dG**	**rA→rU dT→dA**	**rA→rG dT→dC**	**rC→rA dG→dT**	**rC→rU dG→dA**	**rC→rG dG→dC**	**rU→rA dA→dT**	**rU→rC dA→dG**	**rU→rG dA→dC**	**rG→rA dC→dT**	**rG→rC dC→dG**	**rG→rU dC→dA**
Equal	U	39	77	47	4	50	2	1	3	0	17	2	18
	ψ	29	406	39	3	55	3	1	2	0	15	2	21
	m1ψ	46	200	59	5	30	3	2	3	1	19	2	13
	**Relative fold change (ψ to U)**	**0**	**4**	**0**	**0**	**0**	**1**	**0**	**0**	**N/A**	**0**	**0**	**0**
	**Relative fold change (m1ψ to U)**	**0**	**2**	**0**	**0**	**0**	**1**	**1**	**0**	**N/A**	**0**	**0**	**0**
Proportional	U	32	5	27	3	7	1	3	17	2	13	2	2
	ψ	28	42	33	3	11	2	3	14	2	15	2	3
	m1ψ	29	14	37	4	9	3	3	16	2	17	2	1
	**Relative fold change (ψ to U)**	**0**	**7**	**0**	**0**	**1**	**1**	**0**	**0**	**0**	**0**	**0**	**1**
	**Relative fold change (m1ψ to U)**	**0**	**2**	**0**	**0**	**0**	**2**	**0**	**0**	**0**	**0**	**0**	**-1**

Supplementary Table 13. Total and specific error rates of first strand cDNA in RNA sequences synthesized with T7 RNA polymerase in presence of 10 mM, 16 mM and 20 mM rATP. For the reactions with 10 mM or 16 mM rATP, total rNTP concentration in the reaction was 40 mM; for the reaction with 20mM rATP, the total rNTP concentration was 50 mM. $MgCl_2$ concentration in the reactions were matched to the total rNTP in the reactions. Total error rates observed in two different RNA sequences (RNA1 and RNA2) were pooled. The mean and standard deviation were calculated from two independent measurements (n=1 for RNA1and RNA2).

rATP	Base	Total error rate ($\times 10^{-6}$, errors/base)	Substitution (%)	Deletion (%)	Insertion (%)	Total sequenced bases
10 mM	U	64 ± 4	73	12	15	7,820,043
10 mM	ψ	110 ± 20	91	4	5	2,764,788
10 mM	m1ψ	80 ± 3	84	9	7	3,097,951
16 mM	U	45 ± 5	78	10	12	9,146,473
16 mM	ψ	64 ± 1	92	4	4	11,588,053
16 mM	m1ψ	60 ±9	88	6	6	7,360,445
20 mM	U	47 ± 0	71	15	13	19,717,234
20 mM	ψ	66 ± 2	87	6	7	11,101,793
20 mM	m1ψ	48 ± 7	87	8	4	18,970,627

Supplementary Table 14. Substitution error rates of first strand cDNA observed in RNA1/RNA2 (combined; n=2) synthesized with T7 RNA polymerase in presence of 10 mM, 16 mM or 20 mM rATP. The relative fold change was calculated for each substitution as (M—U) / U, where M is the substitution rate on modified RNA and U is the substitution rate on unmodified RNA. N/A, not applicable, as the error rates were rounded to zero in unmodified RNA.

**rATP**	**Uridine analog**	**Substitution error rate (×10^{-6}, errors/base)**											
		**rA→rC dT→dG**	**rA→rU dT→dA**	**rA→rG dT→dC**	**rC→rA dG→dT**	**rC→rU dG→dA**	**rC→rG dG→dC**	**rU→rA dA→dT**	**rU→rC dA→dG**	**rU→rG dA→dC**	**rG→rA dC→dT**	**rG→rC dC→dG**	**rG→rU dC→dA**
10 mM	U	4	5	7	2	7	1	1	8	2	8	1	2
	ψ	4	58	4	8	7	0	3	4	1	5	0	4
	m1ψ	8	15	10	5	7	0	2	7	1	9	1	1
	**Relative fold change (ψ to U)**	**0**	**11**	**0**	**3**	**0**	**-1**	**2**	**-1**	**-1**	**0**	**-1**	**1**
	**Relative fold change (m1ψ to U)**	**1**	**2**	**0**	**2**	**0**	**-1**	**1**	**0**	**-1**	**0**	**0**	**-1**
16 mM	U	2	1	4	1	6	0	4	8	1	5	1	2
	ψ	3	20	6	2	9	0	2	5	1	6	1	4
	m1ψ	4	7	5	4	7	1	6	8	1	7	1	1
	**Relative fold change (ψ to U)**	**1**	**19**	**1**	**1**	**1**	**N/A**	**-1**	**0**	**0**	**0**	**0**	**1**
	**Relative fold change (m1ψ to U)**	**1**	**6**	**0**	**3**	**0**	**N/A**	**1**	**0**	**0**	**0**	**0**	**-1**
20 mM	U	3	2	4	1	6	0	2	8	2	3	1	1
	ψ	3	26	5	1	8	0	1	4	1	4	1	3
	m1ψ	3	6	6	3	6	1	4	4	2	5	0	2
	**Relative fold change (ψ to U)**	**0**	**12**	**0**	**0**	**0**	**N/A**	**-1**	**-1**	**-1**	**0**	**0**	**2**
	**Relative fold change (m1ψ to U)**	**0**	**2**	**1**	**2**	**0**	**N/A**	**1**	**-1**	**0**	**1**	**-1**	**1**

Supplementary Table 15. Total number of substitutions, deletions, and insertions for first-strand cDNA strand synthesis of unmodified and modified RNA used to generate the sequence logos.

			Total reads														
**Enzyme**	**RNA**	**Uridine analog**	**Substitution**	**Deletion**	**Insertion**	**rA→rC dT→dG**	**rA→rU dT→dA**	**rA→rG dT→dC**	**rC→rA dG→dT**	**rC→rU dG→dA**	**rC→rG dG→dC**	**rU→rA dA→dT**	**rU→rC dA→dG**	**rU→rG dA→dC**	**rG→rA dC→dT**	**rG→rC dC→dG**	**rG→rU dC→dA**
T7	RNA1/ RNA2	U	368	62	73	30	36	53	19	55	5	7	59	18	63	8	15
		ψ	265	11	15	10	159	11	21	18	0	8	10	3	14	1	10
		m1ψ	208	22	18	26	48	32	14	21	1	7	23	3	27	2	4
	CLuc	U	1619	121	71	339	289	334	23	231	6	22	210	41	63	24	37
		ψ	1429	42	25	73	1018	94	18	75	10	8	39	14	30	13	37
		m1ψ	67	6	2	7	21	13	5	8	0	4	4	2	3	0	0
	BNT16 2b2	U	322	20	12	33	45	44	9	84	3	9	37	5	41	5	7
		ψ	185	8	0	5	122	11	2	22	1	0	4	1	10	3	4
		m1ψ	362	28	7	42	125	31	19	52	4	11	26	6	31	7	8
SP6	RNA1/ RNA2	U	645	49	74	78	109	130	14	84	7	13	72	8	88	11	31
		ψ	1121	35	83	72	660	104	10	102	5	17	44	8	53	4	42
		m1ψ	926	44	72	116	286	195	26	72	13	20	76	12	83	4	23
	CLuc	U	3925	318	140	764	630	1063	46	374	33	64	483	55	202	34	177
		ψ	2255	73	30	198	1322	262	17	185	5	23	116	10	62	7	48
		m1ψ	8202	399	139	1078	2920	1853	67	562	100	141	719	114	414	49	185

Supplementary Table 16. Oligonucleotide sequence for reverse transcription.

Oligonucleotide	Sequence
Forward primer for RNA1, RNA2, RNA with 6.6% uridine content, RNA with 12.3% uridine content	AGAGTACACGAGTCAGGCTACAGCATC
Reverse primer for RNA1, RNA2, RNA with 6.6% uridine content, RNA with 12.3% uridine content	TACAGTTCACGAGGACCGTCAAGA
Forward primer for CLuc mRNA	CGCCACCATGAAGACCTTAA
Reverse primer for CLuc mRNA	GTCTCCATGCTTTATGTAGC
Forward primer for BNT162b2	TGAACCTGACCACCAGAACA
Reverse primer for BNT162b2	AACTAGCAGAGGTGGTGAGT
Forward primer for RNA with 5.5% uridine content	AAGCCACCTAGAACAACCAC
Reverse primer for RNA with 5.5% uridine content	TTCGTTGGCTTGGACACTTCTG
Forward primer for uridine depleted CLuc RNA	ACCATGAAGACCCTGATCCT
Reverse primer for uridine depleted CLuc RNA	GTGCTTGATGTAGCACTCCT

Supplementary Table 17. *In vitro* transcription template sequences used in this study. Underlined sequence denotes T7 promoter sequence; Underlined bold sequence denotes restriction enzyme sites used for template linearization. For *in vitro* transcription with T3 or SP6 RNAP, the T7 promoter sequence was replaced with either the T3 promoter sequence (AATTAACCCTCACTAAA) or the SP6 promoter sequence (ATTTAGGTGACACTATA).

Construct	Sequence
T7p RNA1	TAATACGACTCACTATAGGGTCTAGAAATAATTTTGTTTAACTTTAGAGTACACG<br>AGTCAGGCTACAGCATCCTCTGGTTCAGACTACTTGATTCATGTGTACCCTATA<br>TGCGAGGATATGTGTATCGTAGAAATTGTCAGGCAGTAACGTTCCGCGAGTTT<br>TAATGGGCGCGCCATGACTCTAAGAGTGATATACCTCCTCGGTCTCGGGCCC<br>GGGGTGTAATTAGCCCAGTTAGACACGATCGCCCGACGTATATTGTTGCTTGG<br>GTATCGTCGCATGCGAAGTATTGCCCAAGGAGACACAACAAGCAACTTATGTT<br>GACTCCCTTCGACCATTAAAATTTGTTAGAACGGACAGAAAGGATGCGCCTTAT<br>AAATGTCCTGTGCAGTGATGAAGCGACCTCAAAACGCTTCATGATCTAACCGA<br>CTCACCTTGCCGTTCCCTCCGCGCCTTAAAACCGGCCGGTCTTGCGAAAAGC<br>GGGAAACGAGTTTACCCACGGATAGCAGGGAATGTTGCGGCTGGCTAGGGAG<br>CATGAAGGTAGATACTCCACGGCTTACCTTTCCGGGGCTCAACATCTAGCCAC<br>AGACCTTTTCGTTAAGCCCACCCCCACTGGATACTGAATCATCAGGGAACCGG<br>ACCCAACCAGTTTGGGCTCGTCCAAGCTTCGGTCTCGTCCCTAAGTGCAAAGA<br>TATGGAAAGAGCAGCATAGGTATATGGATTATTCTTTTACCACTCGTTTCTTAC<br>CGTAACTTACGCAATGGATCACGTGCCGAGGCGGCGGTACAGCTGTTCGAAG<br>GGCTCTGTCGGGAACGCTAACATCCAGCCGGTAAATTCCAAACTAGGGAAAG<br>GACACGCACTGAATTGAATATAGTCGTGAAGGGTGGTGTAAGTCGTGCACAGC<br>CCGCATTAAGTACTAAACAGCGTCCAATCTTGATCTACTTACGGCCTGATGTTC<br>TTCAGCACCTCCTAGCACTGGAGTACTTCGCTATCAATGAGATTAGCACTTTGT<br>ACATGTCATCCAGCCCGAGTCTGGGGTCCGACAATGCGGTCGCCGATTGGTA<br>TCTGCATGTAGTATTAAACGGAGCTGCCGCGGCTGCGGATTATAGTTCATGTC<br>TTGACGGTCCTCGTGAACTGTG**GTTAAC**
T7p RNA2	TAATACGACTCACTATAGGGTCTAGAAATAATTTTGTTTAACTTTAGAGTACACG<br>AGTCAGGCTACAGCATCTTGACACCAGAATATTATGGATTGGACGCTTCCCACT<br>AAATGGAAGACTGTTCGGTCATAAACACTACTAGGAATTCCTCTCCAGTCATCA<br>TGTTCGATCGTCTAGCAGCAATCTCTTCCGATCGATATTTGCGCGTGACTCAGG<br>CGAGCCCATGACAGCTTCTCCCCGTGAGAACCACGACTAGAAGTTATCTGTTG<br>AGCTGCTAGCTTCGTGGCCCGGCCATGGTAGTAGCGGCTCACTCGCGCTAACT<br>TTGCCTGCTCGAGAAAACGGGCGAAACACCCAGCAACACAAGCCACTTAATTT<br>GTTGATAGATAATAAGATCAGGTTATTAGTCGCTCTGCACTTACTTTAAGTGCCA<br>ACTATGCTGTATCGGCCAGGGTGAAAACGGGTGCCGCCACTtCaGTGTGTCGG<br>AGTCTGCTGACGGATTAGGGCACAGACGTATGGTTATATCCTAAGGTAGTGTGT

	CAATGTACTGGGGACAAAGTCAGTGGGCACCGCATCAGGAGTGCAACCTCCG<br>CTAGTACCGACTCGTCAATGCTTTGAGCGATGGCTTGCGCTCCCAAATCCTTAA<br>GCTTTTATGCATTCGGCTCTGGCCCTCAGGCCTGACCTGGAATTTCATCGGAAA<br>CGCCTTAACCGACATTACATCGACACCAAGATCCCGACGCTTCATGCGGAGAC<br>GATAGAGACTCTAACCAAGAATAAAAGGAGTAGTCCCTAATCTACTGAAACGGG<br>GATACCTCAAATCACGGGAATGCGTTACTGACCCGCTATGTGAGGCTCGGATC<br>ACCCTCGTTCTATTGCCTTGTAATCATGGTGGGGCGGCGGAGCGGGATTAGAG<br>GGTGTCCCTAATGTGAGTAGATCTGTAGTAATGATACGTCTCCTCAATATGAGG<br>CGTATTGCAGGTCACAGCACAGGGAGATTTCGGCGCACCCAGCCGAGTTGCCT<br>CCGTCGTTGTTTAGGTATATGCATAACTGCTCACGACAAATACAGCAGAGCCTA<br>CGTTGGGTTATCGAATCCTTGTGGACAAGAAGCTTCTTCATGTCTTGACGGTCC<br>TCGTGAACTGTG**GTTAAC**
T7p 30-nucleotide RNA	CAGTAATACGACTCACTATAGGTGACATACTGACTACAGCCATCCTACCT
T7p 60-nucleotide	CAGTAATACGACTCACTATAGGAAATCCTGATGACGTTGCATACTGCAAAGGTC<br>TTCTGGAGCCGTACAAGGACAGCTGC
T7p CLuc mRNA	TAATACGACTCACTATAGGGAGACCCAAGCTTGGTACCGAGCTCGGATCCGCC<br>ACCATGAAGACCTTAATTCTTGCCGTTGCATTAGTCTACTGCGCCACTGTTCATT<br>GCCAGGACTGTCCTTACGAACCTGATCCACCAAACACAGTTCCAACTTCCTGTG<br>AAGCTAAAGAAGGAGAATGTATTGATAGCAGCTGTGGCACCTGCACGAGAGAC<br>ATACTATCAGATGGACTGTGTGAAAATAAACCAGGAAAAACATGTTGCCGAATG<br>TGTCAGTATGTAATTGAATGCAGAGTAGAGGCCGCAGGATGGTTTAGAACATTC<br>TATGGAAAGAGATTCCAGTTCCAGGAACCTGGTACATACGTGTTGGGTCAAGG<br>AACCAAGGGCGGCGACTGGAAGGTGTCCATCACCCTGGAGAACCTGGATGGA<br>ACCAAGGGGGCTGTGCTGACCAAGACAAGACTGGAAGTGGCTGGAGACATCA<br>TTGACATCGCTCAAGCTACTGAGAATCCCATCACTGTAAACGGTGGAGCTGAC<br>CCTATCATCGCCAACCCGTACACCATCGGCGAGGTCACCATCGCTGTTGTTGA<br>GATGCCAGGCTTCAACATCACCGTCATTGAGTTCTTCAAACTGATCGTGATCGA<br>CATCCTCGGAGGAAGATCTGTAAGAATCGCCCCAGACACAGCAAACAAAGGAA<br>TGATCTCTGGCCTCTGTGGAGATCTTAAAATGATGGAAGATACAGACTTCACTT<br>CAGATCCAGAACAACTCGCTATTCAGCCTAAGATCAACCAGGAGTTTGACGGTT<br>GTCCACTCTATGGAAATCCTGATGACGTTGCATACTGCAAAGGTCTTCTGGAGC<br>CGTACAAGGACAGCTGCCGCAACCCCATCAACTTCTACTACTACACCATCTCCT<br>GCGCCTTCGCCCGCTGTATGGGTGGAGACGAGCGAGCCTCACACGTGCTGCT<br>TGACTACAGGGAGACGTGCGCTGCTCCCGAAACTAGAGGAACCTGCGTTTTGT<br>CTGGACATACTTTCTACGATACATTTGACAAAGCAAGATACCAATTCCAGGGTC<br>CCTGCAAGGAGATTCTTATGGCCGCCGACTGTTTCTGGAACACTTGGGATGTG<br>AAGGTTTCACACAGGAATGTTGACTCTTACACTGAAGTAGAGAAAGTACGAATC<br>AGGAAACAATCGACTGTAGTAGAACTCATTGTTGATGGAAAACAGATTCTGGTT

	GGAGGAGAAGCCGTGTCCGTCCCGTACAGCTCTCAGAACACTTCCATCTACTG<br>GCAAGATGGTGACATACTGACTACAGCCATCCTACCTGAAGCTCTGGTGGTCA<br>AGTTCAACTTCAAGCAACTGCTCGTCGTACATATTAGAGATCCATTCGATGGTA<br>AGACTTGCGGTATTTGCGGTAACTACAACCAGGATTTCAGTGATGATTCTTTTG<br>ATGCTGAAGGAGCCTGTGATCTGACCCCCAACCCACCGGGATGCACCGAAGAA<br>CAGAAACCTGAAGCTGAACGACTCTGCAATAGTCTCTTCGCCGGTCAAAGTGAT<br>CTTGATCAGAAATGTAACGTGTGCCACAAGCCTGACCGTGTCGAACGATGCAT<br>GTACGAGTATTGCCTGAGGGGACAACAGGGTTTCTGTGACCACGCATGGGAGT<br>TCAAGAAAGAATGCTACATAAAGCATGGAGACACCCTAGAAGTACCAGATGAAT<br>GCAAATAG**GCGGCCGC**
T7p<br>BNT162b2<br>mRNA	TAATACGACTCACTATAGGAGAATAAACTAGTATTCTTCTGGTCCCCACAGACT<br>CAGAGAGAACCCGCCACCATGTTCGTGTTCCTGGTGCTGCTGCCTCTGGTGTC<br>CAGCCAGTGTGTGAACCTGACCACCAGAACACAGCTGCCTCCAGCCTACACCA<br>ACAGCTTTACCAGAGGCGTGTACTACCCCGACAAGGTGTTCAGATCCAGCGTG<br>CTGCACTCTACCCAGGACCTGTTCCTGCCTTTCTTCAGCAACGTGACCTGGTTC<br>CACGCCATCCACGTGTCCGGCACCAATGGCACCAAGAGATTCGACAACCCCGT<br>GCTGCCCTTCAACGACGGGGTGTACTTTGCCAGCACCGAGAAGTCCAACATCA<br>TCAGAGGCTGGATCTTCGGCACCACACTGGACAGCAAGACCCAGAGCCTGCT<br>GATCGTGAACAACGCCACCAACGTGGTCATCAAAGTGTGCGAGTTCCAGTTCT<br>GCAACGACCCCTTCCTGGGCGTCTACTACCACAAGAACAACAAGAGCTGGATG<br>GAAAGCGAGTTCCGGGTGTACAGCAGCGCCAACAACTGCACCTTCGAGTACGT<br>GTCCCAGCCTTTCCTGATGGACCTGGAAGGCAAGCAGGGCAACTTCAAGAACC<br>TGCGCGAGTTCGTGTTTAAGAACATCGACGGCTACTTCAAGATCTACAGCAAGC<br>ACACCCCTATCAACCTCGTGCGGGATCTGCCTCAGGGCTTCTCTGCTCTGGAA<br>CCCCTGGTGGATCTGCCCATCGGCATCAACATCACCCGGTTTCAGACACTGCT<br>GGCCCTGCACAGAAGCTACCTGACACCTGGCGATAGCAGCAGCGGATGGACA<br>GCTGGTGCCGCCGCTTACTATGTGGGCTACCTGCAGCCTAGAACCTTCCTGCT<br>GAAGTACAACGAGAACGGCACCATCACCGACGCCGTGGATTGTGCTCTGGATC<br>CTCTGAGCGAGACAAAGTGCACCCTGAAGTCCTTCACCGTGGAAAAGGGCATC<br>TACCAGACCAGCAACTTCCGGGTGCAGCCCACCGAATCCATCGTGCGGTTCCC<br>CAATATCACCAATCTGTGCCCCTTCGGCGAGGTGTTCAATGCCACCAGATTCG<br>CCTCTGTGTACGCCTGGAACCGGAAGCGGATCAGCAATTGCGTGGCCGACTAC<br>TCCGTGCTGTACAACTCCGCCAGCTTCAGCACCTTCAAGTGCTACGGCGTGTC<br>CCCTACCAAGCTGAACGACCTGTGCTTCACAAACGTGTACGCCGACAGCTTCG<br>TGATCCGGGGAGATGAAGTGCGGCAGATTGCCCCTGGACAGACAGGCAAGAT<br>CGCCGACTACAACTACAAGCTGCCCGACGACTTCACCGGCTGTGTGATTGCCT<br>GGAACAGCAACAACCTGGACTCCAAAGTCGGCGGCAACTACAATTACCTGTAC<br>CGGCTGTTCCGGAAGTCCAATCTGAAGCCCTTCGAGCGGGACATCTCCACCGA<br>GATCTATCAGGCCGGCAGCACCCCTTGTAACGGCGTGGAAGGCTTCAACTGCT<br>ACTTCCCACTGCAGTCCTACGGCTTTCAGCCCACAAATGGCGTGGGCTATCAG

	CCCTACAGAGTGGTGGTGCTGAGCTTCGAACTGCTGCATGCCCCTGCCACAGT<br>GTGCGGCCCTAAGAAAAGCACCAATCTCGTGAAGAACAAATGCGTGAACTTCA<br>ACTTCAACGGCCTGACCGGCACCGGCGTGCTGACAGAGAGCAACAAGAAGTT<br>CCTGCCATTCCAGCAGTTTGGCCGGGATATCGCCGATACCACAGACGCCGTTA<br>GAGATCCCCAGACACTGGAAATCCTGGACATCACCCCTTGCAGCTTCGGCGGA<br>GTGTCTGTGATCACCCCTGGCACCAACACCAGCAATCAGGTGGCAGTGCTGTA<br>CCAGGACGTGAACTGTACCGAAGTGCCCGTGGCCATTCACGCCGATCAGCTGA<br>CACCTACATGGCGGGTGTACTCCACCGGCAGCAATGTGTTTCAGACCAGAGCC<br>GGCTGTCTGATCGGAGCCGAGCACGTGAACAATAGCTACGAGTGCGACATCCC<br>CATCGGCGCTGGAATCTGCGCCAGCTACCAGACACAGACAAACAGCCCTCGG<br>AGAGCCAGAAGCGTGGCCAGCCAGAGCATCATTGCCTACACAATGTCTCTGGG<br>CGCCGAGAACAGCGTGGCCTACTCCAACAACTCTATCGCTATCCCCACCAACT<br>TCACCATCAGCGTGACCACAGAGATCCTGCCTGTGTCCATGACCAAGACCAGC<br>GTGGACTGCACCATGTACATCTGCGGCGATTCCACCGAGTGCTCCAACCTGCT<br>GCTGCAGTACGGCAGCTTCTGCACCCAGCTGAATAGAGCCCTGACAGGGATC<br>GCCGTGGAACAGGACAAGAACACCCAAGAGGTGTTCGCCCAAGTGAAGCAGA<br>TCTACAAGACCCCTCCTATCAAGGACTTCGGCGGCTTCAATTTCAGCCAGATTC<br>TGCCCGATCCTAGCAAGCCCAGCAAGCGGAGCTTCATCGAGGACCTGCTGTTC<br>AACAAAGTGACACTGGCCGACGCCGGCTTCATCAAGCAGTATGGCGATTGTCT<br>GGGCGACATTGCCGCCAGGGATCTGATTTGCGCCCAGAAGTTTAACGGACTGA<br>CAGTGCTGCCTCCTCTGCTGACCGATGAGATGATCGCCCAGTACACATCTGCC<br>CTGCTGGCCGGCACAATCACAAGCGGCTGGACATTTGGAGCAGGCGCCGCTC<br>TGCAGATCCCCTTTGCTATGCAGATGGCCTACCGGTTCAACGGCATCGGAGTG<br>ACCCAGAATGTGCTGTACGAGAACCAGAAGCTGATCGCCAACCAGTTCAACAG<br>CGCCATCGGCAAGATCCAGGACAGCCTGAGCAGCACAGCAAGCGCCCTGGGA<br>AAGCTGCAGGACGTGGTCAACCAGAATGCCCAGGCACTGAACACCCTGGTCAA<br>GCAGCTGTCCTCCAACTTCGGCGCCATCAGCTCTGTGCTGAACGATATCCTGA<br>GCAGACTGGACCCTCCTGAGGCCGAGGTGCAGATCGACAGACTGATCACAGG<br>CAGACTGCAGAGCCTCCAGACATACGTGACCCAGCAGCTGATCAGAGCCGCC<br>GAGATTAGAGCCTCTGCCAATCTGGCCGCCACCAAGATGTCTGAGTGTGTGCT<br>GGGCCAGAGCAAGAGAGTGGACTTTTGCGGCAAGGGCTACCACCTGATGAGC<br>TTCCCTCAGTCTGCCCCTCACGGCGTGGTGTTTCTGCACGTGACATATGTGCC<br>CGCTCAAGAGAAGAATTTCACCACCGCTCCAGCCATCTGCCACGACGGCAAAG<br>CCCACTTTCCTAGAGAAGGCGTGTTCGTGTCCAACGGCACCCATTGGTTCGTG<br>ACACAGCGGAACTTCTACGAGCCCCAGATCATCACCACCGACAACACCTTCGT<br>GTCTGGCAACTGCGACGTCGTGATCGGCATTGTGAACAATACCGTGTACGACC<br>CTCTGCAGCCCGAGCTGGACAGCTTCAAAGAGGAACTGGACAAGTACTTTAAG<br>AACCACACAAGCCCCGACGTGGACCTGGGCGATATCAGCGGAATCAATGCCA<br>GCGTCGTGAACATCCAGAAAGAGATCGACCGGCTGAACGAGGTGGCCAAGAA<br>TCTGAACGAGAGCCTGATCGACCTGCAAGAACTGGGGAAGTACGAGCAGTACA

	TCAAGTGGCCCTGGTACATCTGGCTGGGCTTTATCGCCGGACTGATTGCCATC<br>GTGATGGTCACAATCATGCTGTGTTGCATGACCAGCTGCTGTAGCTGCCTGAA<br>GGGCTGTTGTAGCTGTGGCAGCTGCTGCAAGTTCGACGAGGACGATTCTGAGC<br>CCGTGCTGAAGGGCGTGAAACTGCACTACACATGATGACTCGAGCTGGTACTG<br>CATGCACGCAATCCTAGCTGCCCCTTTCCCGTCCTGGGTACCCCGAGTCTCCC<br>CCGACCTCGGGTCCCAGGTATGCTCCCACCTCCACCTGCCCCACTCACCACCT<br>CTGCTAGTTCCAGACACCTCCCAAGCACGCAGCAATGCAGCTCAAAACGCTTA<br>GCCTAGCCACACCCCCACGGGAAACAGCAGTGATTAACCTTTAGCAATAAACG<br>AAAGTTTAACTAAGCTATACTAACCCCAGGGTTGGTCAATTTCGTGCCAGCCAC<br>ACCCTGGACCTAGCGCGGCCG**GCTAGC**
T7p RNA with 5.5% uridine content	TAATACGACTCACTATAGGGTCTAGAGCGTCGGCAAGCCACCTAGAACAACCA<br>CGGCACGCGAACCAACAGCGGCAACGCCGAACGGCCACGCGACGAACTGAG<br>CACCACCACAAGACCAAGCCAACGAGGAACACCGAACAACCGGTCACTGAACG<br>CAACGGCCTAGGCGCGCACACCAATAAGACGGATCAAGCCAGCGCATGACTG<br>CGCCGGACAACGCCACGGACGACGAACAGGTCCACCGGAGTACGACTATGCC<br>ATACCAGCAGGACAGCGGAGGCGACCAACAACAACAGGAGCGGCAGCCGGCA<br>GACGAATTGGACCGACCGGACATGGCCACATGCGATTGAGCCGGAACCGCGC<br>GCACTTGACAGTCAGCAACCGGCTCCAACAGTCCGGCACCATGAGAGAACCG<br>GCAAGCCGGCCTTGCCACAGGAACGCAGAGAGCGGAGCAACACAAGCCGACG<br>CCAAGCCAACCGCGAACCAGCCAGGCGCCGCCGGCAACAGGAAGGTCGCAC<br>GAGCCAACATATGGACCGGCCGGAACACTAAGGACCAAGAGGCCACTGGCGC<br>GGAACGGAGAACCAGGCACCAAGCAAGGAACAGAGAGGCATAGGAACCGAAG<br>GCGCCATAGCCGGCGAGACGACCAGGAACGAACTGACAGCCAACGCTAGAGG<br>AACGTGACGATGGACGAACGTAGACACGGCGGCGCAACGCCGCGGCGGAGT<br>CCGGAACCGGCGCTAAGGTCCACACGGCAGAACCGGCCAGGCCGAGGCAAC<br>CGCCGCCACACGACCACAGCAGCCAGAAGGATAGCGCGCGGCCATAACAGGA<br>ACAGCAAGGACAGGCGCCACGCAGGACCGCAACTGAGCAACCGGCAACCACC<br>TCCACCGAAGCCGCGAAGCAGAGGCCGACGACGGCACACGCGCCATGAGCG<br>GCACAACCAGCCAGAAGGCCAACGCGCCAAGGCGACCGAAGCCAACTACAGA<br>AGTGTCCAAGCCAACGAAGCCAACCGGCAGAAGAGAGCAGG**GTTAAC**
T7p RNA with 6.6% uridine content	TAATACGACTCACTATAGGGTCTAGAAATAATTTTGTTTAACTTTAGAGTACACG<br>AGTCAGGCTACAGCATCCTCCAGGCAGCCACACAACGGAGGCAAGAGCGGCA<br>AGCGCAACAACGCGCTCCGCGACGAGCGCGCAGAGTATCGGCAGATTCCACC<br>GGACGGAGCACGAGGACGAAGCGACCGGTCCACACGGCTGGAGGACGCAGC<br>GCCAAGCTCGGTGCCGACCGAAGCCGAGCGAGAGGAACGCCGAGAAGCAAG<br>CCGCGACTCCACAGAGCCGCCAGCCGAAGAAGACGCACGCCAGGAGGAACAG<br>GAAGCGAGCGGAAGGTCAACGCACCAAGGCCAGCAGGACTAAGCCGGCAGAC<br>GGCGGCCGCCACCACCAAGCGAACAATGATACACGAGAACGGACCGGCAACA<br>TGGACACGGCGTAGTCCACAGAGCCACAAGGCAGTGGTGCGAACCGGCAACG<br>GAGAACAATACCGGAAGAGCACAACAGAGAGGAGCTGGCCGGCACACGAGCG

	CGACGCGGCACCGGCGCGCCACACCAGCCGAAGGCAGCCAGCACCAACGAG<br>CGCGGCGCACCGACTGACGGCGAGAACAGACCGAAGGCCGGCGGCCACCAC<br>GGACGCGGACCAGAAGACCGGATGGCAGGCGACAACGACGGCTCGCCTCCAA<br>CAAGAAGCGGCGGACCGGCGGAAGCGACAGGCGCCGCACGTGAACACAACA<br>ACTGCACGGCATGCGGCGAACGGCCAAGCGAGACGACGCGCTCCAAGAGCCA<br>ACGATAGAATGCTACGGCAACCGAGCAGAGGCAGAGCGCCAGCGCAACACAC<br>CAGAAGAACGCGACGAGAGAGACGGACGCCGGCGCGGACCATTGGAGGCGG<br>AGAACCGACAACCGCAATGCGGAGATCAAGGCGGACCGAACGACAACAGCAG<br>AGCCACCAACTGCGCCGTGGTCGCAACCAACCTAACCAGCAACAACAGGCACC<br>GCCGCGGCGACACAGGACGAGGCCGCCGAACAACGATAACGCGACACCAGG<br>CACACACACGAACCGCGGAAGGACCACAGCGACAGCCATTCATGTCTTGACGG<br>TCCTCGTGAACTGTG**GTTAAC**
T7p RNA with 12.3% uridine content	TAATACGACTCACTATAGGGTCTAGAAATAATTTTGTTTAACTTTAGAGTACACG<br>AGTCAGGCTACAGCATCCTCACACACCAATGATCCAATAACCTCGAAGGCGAC<br>ACCGCATGTCGCCACCAGCCGTGAACGACGGAAGAATAACCGCAGCGAATAC<br>GGACATCAAGAGGCACAGAACCTACGAAGCAACACGAACGCACACCATAACTT<br>CAGCGGCAACGAAGCCGACTGAGGTCCGGCCGGCAACCAAGGCCGAAGTTCA<br>CTAAGCCTCGTAACGGCAGCCGTATAGGACCTCGCCGAGCACCAGACGACAC<br>CAAGAGAAGGCAGCGACCAACTGCGTCGGCCGGCGAACTGCCGAAGCTAAGG<br>CACAGACCGGTCGCCAGGCCGCAGACCGCGGAACCAACAAGAGGCAGCGCTA<br>GGCCATCTCAGCACCACCAAGACATTGATATTAACAGAACGCGGAATCAAGCG<br>TACGATACCAACGACAAGTGTGACGAACGTGCGTCTGAATGGCACATAAGGTC<br>GCCGGTAGCGAACGCCGAACCGAACCACCTACCGCAACCAACCGACACCATA<br>GGATGTCACAGACGACTCGCAACGGCTAGACCTTACCAACACAGGAACATCAC<br>AAGAATCATAACACGAGCCACAAGAAGCCGATGCAAGCGGAGCCGGACCGCG<br>GCACCAAGGCGCGGAACACGACGCCAGGCCAAGCAGAAGCTCGCCGGCGGC<br>CAGCAAGGTCCACAGCGGCGCGGCCACCAGTGCACTCTATGTTGAGAGTGGA<br>CCAACGTTGACGCCGAAGGCTCACAATGGCTGAGCGCTGTAAGTGGCGTCGC<br>GCGCCAGGTTGCCACACCAGGAACAGACGCAACAACCACGAGCGGCCGTACA<br>AGCATAGAGCCGTGCCTGAATGGTTGATCCAGTAGACGGCCACAAGGCAGGT<br>GGCGGTCGAACCGAATGGCTAGGTCCGGCTCACAAGGATAACACGGCTACAC<br>CTGAACGCCGCCGCAGGCGCAACGACGAAGCCGTAGCAACGCAGAGAGATAA<br>CGCAACGGCCAGGCAAGAGATACACAATTTCATGTCTTGACGGTCCTCGTGAA<br>CTGTG**GTTAAC**
T7p Uridine-depleted CLuc mRNA	TAATACGACTCACTATAGGGAGACCCAAGCUUGGUACCGAGCUCGGAUCCGCCACCA<br>UGAAGACCCUGAUCCUGGCCGUGGCCCUGGUGUACUGCGCCACCGUGCACUGCCAGG<br>ACUGCCCAUACGAACCAGACCCCCCGAACACCGUGCCAACCAGCUGCGAGGCCAAGGA<br>AGGCGAGUGCAUCGACAGCAGCUGCGGCACCUGCACCAGAGACAUCCUGAGCGACGG<br>CCUGUGCGAGAACAAGCCGGGAAAGACAUGCUGCCGGAUGUGCCAGUACGUGAUCGA<br>GUGCAGAGUGGAGGCCGCAGGAUGGUUCCGGACCUUCUACGGCAAGAGAUUCCAGUU

	CCAAGAGCCCGGCACAUACGUGCUGGGCCAGGGAACCAAGGGCGGCGACUGGAAAGU<br>GAGCAUCACCCUGGAGAACCUCGACGGCACCAAAGGCGCCGUGCUGACAAAGACAAG<br>ACUGGAAGUCGCCGGCGACAUCAUCGACAUCGCGCAGGCCACCGAGAACCCCAUCACC<br>GUGAACGGAGGCGCCGACCCCAUAAUCGCCAACCCCUACACAAUCGGCGAAGUGACAA<br>UCGCCGUCGUGGAAAUGCCAGGCUUCAACAUCACAGUGAUCGAAUUCUUCAAGCUGA<br>UCGUGAUCGACAUACUGGGCGGACGGAGCGUGCGCAUCGCCCCAGACACCGCGAACA<br>AGGGCAUGAUCAGCGGCCUGUGCGGAGACCUGAAGAUGAUGGAGGACACCGACUUCA<br>CCAGCGACCCCGAGCAGCUGGCCAUCCAGCCAAAAAUCAACCAGGAAUUCGACGGCUG<br>CCCCCUGUACGGAAACCCCGACGACGUGGCCUACUGCAAAGGCCUGCUCGAGCCGUA<br>CAAGGACAGCUGCAGAAACCCCAUCAACUUCUACUACUACACCAUCAGCUGCGCCUUC<br>GCCAGGUGCAUGGGCGGCGACGAAAGAGCCAGCCACGUCCUGCUGGACUACAGAGAA<br>ACCUGCGCCGCCCCGGAGACACGGGGCACCUGCGUGCUGAGCGGCCACACCUUCUAC<br>GACACAUUCGACAAGGCACGGUACCAGUUCCAGGGCCCAUGCAAGGAGAUCCUGAUG<br>GCCGCCGACUGCUUCUGGAACACCUGGGACGUGAAGGUGAGCCACAGAAACGUCGAC<br>AGCUACACAGAGGUGGAGAAGGUGAGAAUCAGAAAACAGAGCACAGUGGUGGAACUG<br>AUCGUGGACGGCAAGCAAAUUCUGGUGGGCGGCGAAGCCGUGAGCGUGCCAUACAGC<br>AGCCAAAACACCAGCAUCUACUGGCAGGACGGCGACAUCCUGACAACCGCCAUCCUGC<br>CCGAGGCACUGGUGGUGAAGUUCAACUUCAAACAGCUGCUGGUGGUCCACAUCAGAG<br>ACCCCUUCGACGGCAAGACAUGCGGAAUCUGCGGCAACUACAACCAGGACUUCAGCGA<br>CGACAGCUUCGACGCCGAGGGCGCCUGCGACCUGACCCCCAACCCGCCCGGCUGCAC<br>CGAGGAACAGAAGCCAGAGGCCGAAAGACUGUGCAACAGCCUCUUCGCCGGACAGAG<br>CGACCUGGACCAGAAGUGCAACGUGUGCCACAAACCGGACAGAGUGGAACGGUGCAU<br>GUACGAAUACUGCCUGCGGGGCCAGCAGGGAUUCUGCGACCACGCCUGGGAGUUCAA<br>GAAGGAGUGCUACAUCAAGCACGGCGACACCCUGGAGGUGCCAGACGAGUGCAAGUA<br>G**<u>GCGGCCGC</u>**

References

1 Crooks, G. E., Hon, G., Chandonia, J. M. & Brenner, S. E. WebLogo: a sequence logo generator. *Genome Res* **14**, 1188-1190, doi:10.1101/gr.849004 (2004).

2 Potapov, V. *et al.* Base modifications affecting RNA polymerase and reverse transcriptase fidelity. *Nucleic Acids Res* **46**, 5753-5763, doi:10.1093/nar/gky341 (2018).
